# Comparative study of serum inflammatory mediators and immunoglobulin levels in children with severe asthma infected by viruses versus bacteria

**DOI:** 10.5937/jomb0-60933

**Published:** 2026-04-15

**Authors:** Xiaoxia Zhang, Guojiang Jian, Xiaoqun Zhong, Mao Li, Shifang Wan, Huan Liu, Xiangrui Chen

**Affiliations:** 1 Luzhou People's Hospital, Department of General Pediatrics, Luzhou, Sichuan, China

**Keywords:** bacterial infection, viral infection, bronchial asthma, inflammatory factors, immunoglobulin, bakterijska infekcija, virusna infekcija, bronhijalna astma, inflamatorni faktori, imunoglobulin

## Abstract

**Background:**

By tracking fluctuations in inflammatory mediators and immunoglobulins (Igs) in bacterialversus viral-induced severe asthma (SA) cases, this research seeks to identify microbial-specific immune characteristics to guide individualized treatment.

**Methods:**

This analysis included 100 children hospitalized between March 2023 and April 2025 for severe acute SA exacerbations with confirmed single-pathogen infections (50 viral vs. 50 bacterial). Serum samples collected preand post-treatment were analyzed for inflammatory mediators (Th1/Th2/Th17 cytokines and TNF-a) and Ig profiles (total IgE and IgG subclasses). Comparative analysis was performed to identify intergroup differences.

**Results:**

At baseline, the viral group showed higher Th1/Th17 co-activation responses, whereas the bacterial group had elevated Th2 cytokines/TNF-a (P &lt; 0.05). Therapeutic intervention reduced all cytokine subsets, though Th1/Th17 co-activation suppression was more marked in the viral group (P&lt; 0.05), and Th2 decay lagged in the bacterial group. Besides, viral cases had elevated IgG1/IgG3; bacterial cases showed higher IgG2 (P&lt; 0.05). After treatment, the levels of IgG1 and IgG3 in the virus group continued to increase, but IgG2 did not change.

**Conclusions:**

Viral SA infections predominantly trigger Th1/Th17 co-activation-mediated inflammation and higher IgG1/IgG3. In contrast, bacterial infections favor a Th2-dominant profile with IgG2 elevation, resulting in slower inflammation resolution.

## Abstract (sr)

**Uvod:**

Pracenjem fluktuacija inflamatornih medijatora i imunoglobulina (Ig) kod slučajeva teške astme (SA) izazvane bakterijama u odnosu na viruse, ovo istraživanje ima za cil j da identifikuje specifične imunološke karakteristike mikroba kako bi se usmerio individualizovani tretman.

**Metode:**

Ova analiza je obuhvatila 100 dece hospitalizovane između marta 2023. i aprila 2025. godine zbog teških akut-nih egzacerbacija SA sa potvrđenim infekcijama jednim patogenom (50 virusnih u odnosu na 50 bakterijskih). Uzorci seruma prikupljeni pre i posle lečenja analizirani su na inflamatorne medijatore (Th1/Th2/Th17 citokine i TNF-a) i Ig profile (ukupne podklase IgE i IgG). Komparativna analiza je sprovedena kako bi se identifikovale razlike između grupa.

**Rezultati:**

Na početku, virusna grupa je pokazala veće odgovore koaktivacije Th1/Th17, dok je bakterijska grupa imala povišene Th2 citokine/TNF-a (P&lt;0,05). Terapeutska intervencija je smanjila sve podskupove citokina, iako je supresija koaktivacije Th1/Th17 bila izraženija u virusnoj grupi (P&lt; 0,05), a opadanje Th2 je zaostalo u bakterijskoj grupi. Pored toga, virusni slučajevi su imali povišen IgG1/IgG3; bakterijski slučajevi su pokazali viši IgG2 (P&lt; 0,05). Nakon tretmana, nivoi IgG1 i IgG3 u virusnoj grupi su nastavili da rastu, ali IgG2 se nije promenio.

**Zaključak:**

Virusne SA infekcije pretežno pokreću upalu posredovanu koaktivacijom Th1/Th17 i viši IgG1/IgG3. Nasuprot tome, bakterijske infekcije favorizuju Th2-dominantan profil sa povišenjem IgG2, što rezultira sporijim rešavanjem upale.

## Introduction

Asthma ranks as the most prevalent chronic inflammatory airway disorder among children. Notably, 10% to 15% of pediatric cases progress to severe asthma (SA), which involves recurrent acute exacerbations and often proves resistant to standard therapies, necessitating higher doses of corticosteroids [1]. Globally, healthcare institutions have identified SA as a critical public health challenge in pediatric respiratory care, with significant implications for patient well-being and familial socioeconomic strain [2]. SA exacerbations are frequently induced by respiratory pathogens, particularly viruses (60-80% of acute cases) [3]. The cornerstone of clinical intervention remains pathogen-targeted anti-infective therapy [4]. In clinical practice, bacterial culture remains the primary diagnostic tool for pathogens. However, standard bacterial culture requires 24-48 hoursfor results, delaying critical treatment [5]. Procedural errors during sampling could also carry iatrogenic infection risks, particularly in immunodeficient SA children [6]. Literature reports an average diagnostic delay of 36 hours in pediatric SA emergencies [7]. Prompt pathogen identification is critical to guide precise therapeutic decisions in SA.

Recent studies have elucidated key mechanisms underlying SA inflammation. Th2 inflammation has been confirmed as central to allergic SA. Conversely, viral infections can exacerbate airway hyperresponsiveness by triggering Th1/Th17 co-activation responses via epithelial pattern recognition receptor (PRR) activation [8]. Infections caused by bacteria stimulate the innate immune response by releasing endotoxins (e.g., lipopolysaccharide [LPS]) or peptidoglycan. This promotes the secretion of pro-inflammatory factors while impairing regulatory T cells (Tregs), contributing to uncontrolled inflammation [9]. Immunoglobulin (Ig) E (IgE) is a critical driver of allergic reactions, with elevated levels directly correlating to SA severity. Meanwhile, IgG subclasses provide airway protection through two mechanisms, either by neutralizing pathogens or modulating IgE-mediated immune effects [10]. Hence, serum inflammatory and Ig testing may aid pathogen diagnosis in SA. However, further research is needed due to the scarcity of published data.

Focusing on SA cases caused by diverse pathogens, this research examines fluctuations in Th1/Th2/Th17 cytokines and Ig levels. The study aims to uncover links between inflammatory-immune signatures and clinical severity, offering insights for early risk stratification and precision treatment. Additionally, dysregulation of Ig subclasses could reveal novel immune modulation targets, enabling tailored immunoregulatory therapy. This advancement holds significant promise for reducing acute SA exacerbations and severe disease progression, ultimately improving long-term outcomes in pediatric patients.

## Materials and methods

### Research population and time frame

Pediatric SA patients presenting to our facility during the period spanning March 2023 to April 2025 were selected for this investigation. This study has been approved by the Ethics Committee of Luzhou People's Hospital, with parental/guardian consent acquired for all study participants.

### Criteria for patient cohort

Eligibility requirements: (1) Aged 3-14 years; (2) Confirmed SA diagnosis [11]; (3) Laboratory-confirmed mono-infection: Virus: Nasal/throat swab or sputum nucleic acid testing-positive ( 10] copies/mL) with no bacterial growth. Bacteria: Sputum/blood culture yielding pathogenic bacteria (VITEK 2-verified), excluding viral coinfection.

Disqualification factors: (1) Congenital airway malformations, bronchopulmonary dysplasia, or congenital heart disease; (2) Recent immunosuppressive/biologic drug use ( 30 days); (3) Immunodeficiency diagnoses or regular immunomodulators; (4) Non-asthma-related wheezing; (5) Non-compliant with study procedures.

### Grouping

The study's sample size determination relied on initial pilot study findings. IL-17A was selected as the primary endpoint for sample size estimation based on pilot data showing its significant differential expression (Δ=12 pg/mL) between infection types. To achieve α=0.05 and β=0.2, calculations indicated the need for 44 patients per study arm. After adjusting for possible 10% participant attrition, the final enrollment target was set at 50 children per diagnostic category. Consequently, the research included 100 participants in total, evenly distributed between viral (n = 50) and bacterial (n = 50) groups based on laboratory-confirmed diagnoses. Laboratory staff performing biomarker assays were blinded to group assignment to minimize bias.

### Intervention strategies

Standard management for pediatric SA acute exacerbations was administered to all subjects, including oxygenation, bronchodilators, and glucocorticoids (methylprednisolone 1-2 mg/kg/day IV for 3-5 days, tapered based on clinical response). Treatment was tailored to etiology: viral cases received supportive measures (with oseltamivir for influenza-positive patients), while bacterial infections were treated with sensitivity-directed antibiotics like amoxicillin-clavulanate or cephalosporin derivatives.

### Laboratory methodology

Pre-treatment and post-treatment morning fasting venous blood specimens (4 mL each) were acquired from hospitalized pediatric subjects for subsequent laboratory testing. (i) Quantification of Th1/Th2/Th17-associated cytokines (IFN-γ, IL-4, IL-5, IL-13, IL-17A) was conducted with a high-sensitivity multiplex immunoassay kit (Meso Scale Discovery, Catalog #K15067G-1, Lot #20230315). Following collection, blood samples were immediately placed on ice and centrifuged (3,000 X g, 10 min) for separation. Analysis was carried out on an MSD SECTOR S600 electrochemiluminescence platform, with technical triplicates and internal controls included per plate. Concentration values were derived using Discovery Workbench 4.0 software. Assay validation required quality controls at low, medium, and high concentrations (CV <15%), with intra-assay CV maintained at 10%. (ii) Measurement of TNF-α was conducted using a human TNF-α ELISA kit (R&D Systems, Catalog #HSTA00E, Version 3.0). Serum samples were processed consistently with earlier steps. The assay procedure included standard curve preparation, incubation with biotinylated antibodies, and HRP-based chromogenic detection (450 nm absorbance). Concentrations were derived from the standard curve, with blank controls showing absorbance <0.1. Samples beyond the detectable range were diluted 1: 10 and reanalyzed. (iii) An automated biochemistry analyzer (BS-5800, Mindray) measured immunoglobulins (Igs) (including IgE, IgG1-IgG4). Serum samples were processed as described above and directly loaded for testing. The instrument performed automatic calibration, and Ig concentrations were determined via nephelometry. Prior to daily operation, high and low value quality controls (CV <5%) were run.

### Statistical analysis

The statistical evaluation was carried out in SPSS 34.0. Count data [n (%)] underwent chi-square testing. Continuous data distribution was verified by Shapiro-Wilk testing: Normally distributed measures (P>0.05) appeared as means ± SD, analyzed through t-tests (independent for inter-group and paired for within-group comparisons). Non-parametric data (P<0.05) displayed as [M (P25, P75)] received non-parametric testing (Mann-Whitney U for between-group, Wilcoxon signed-rank test for within-group analyses). Statistical comparisons employed ANOVA for longitudinal cytokine changes (with Bonferroni correctionfor multiple comparisons). A significance threshold of P<0.05 was applied for all statistical comparisons.

## Results

### Comparative assessment of patient baseline data

Evaluations of patient age, sex ratio, and SA duration revealed comparable profiles across groups (P>0.05, Table 1), supporting equitable cohort matching for subsequent analysis.

**Table 1:** Clinical data of the children.

	Bacterial group (n = 50)	Viral group (n = 50)	t (χ^2^)	P
Age	8.32±3.43	9.40±3.67	1.520	0.132
Sex			0.679	0.410
Boys	33 (66.00)	29 (58.00)		
Girls	17 (34.00)	21 (42.00)		
SA Duration (months)	4.22±1.02	4.44±1.15	1.016	0.312

### Comparative analysis of Th1 and Th17 cytokine expression

Baseline IFN-γ and IL-17A concentrations were higher in viral versus bacterial subjects (P<0.05). Post-therapy measurements revealed an opposite trend, with viral cases displaying lower cytokine levels than bacterial ones (P<0.05). Post-treatment measurements indicated a significant drop in IFN-γ and IL-17A from baseline values in both cohorts (P<0.05). Further analysis of the differences between groups demonstrated that the viral group had a larger decrease in these cytokines than the bacterial cohort (P<0.05, Table 2).

**Table 2:** Comparison of Th1/Th17 co-activation type cytokines. Note: * indicates P < 0.05 compared with before-treatment.

		Bacterial group<br>(n = 50)	Viral group (n = 50)	t	P
IFN-g (pg/mL)	Before treatment	124.33±22.19	158.63±23.92	7.434	<0.001
	After treatment	76.80±25.21*	64.70±21.08*	2.604	0.011
	Before - after	47.52±34.22	93.93±30.52	7.156	<0.001
IL-17A (pg/mL)	Before treatment	45.10±8.66	55.41±9.59	5.644	<0.001
	After treatment	25.60±7.33*	21.33±7.43*	2.898	0.005
	Before - after	19.50±11.01	34.09±11.65	6.436	<0.001

### Comparative analysis of Th2 cytokine variations

The viral group had lower baseline IL-4, IL-5, and IL-13 than the bacterial group (P<0.05), but after treatment, the differences disappeared (P>0.05). Both groups showed decreased IL-4, IL-5, and IL-13 levels post-treatment (P<0.05), with a more significant drop in the viral group (P<0.05, Table 3).

**Table 3:** Comparison of Th2-type cytokines. Note: * indicates P < 0.05 compared with before-treatment.

		Bacterial group (n=50)	Viral group (n=50)	t	P
IL-4 (pg/mL)	Before treatment	42.08±6.09	50.60±7.72	4.332	<0.001
	After treatment	26.87±4.65*	27.19±6.43*	0.775	0.287
	Before - after	15.21±7.55	23.41±10.29	4.545	<0.001
IL-5 (pg/mL)	Before treatment	34.55±6.17	38.78±5.66	3.567	<0.001
	After treatment	20.29±5.52*	20.61±5.72*	0.284	0.777
	Before - after	14.26±8.46	18.17±8.67	2.281	0.025
IL-13 (pg/mL)	Before treatment	65.90±7.45	75.23±8.58	5.806	<0.001
	After treatment	30.99±6.82*	31.42±6.10*	0.334	0.739
	Before - after	34.91±10.14	43.80±11.36	4.131	<0.001

### Comparison of pro-inflammatory factor variations

Compared to the bacterial group, the viral group showed lower TNF-α levels both at baseline and post-treatment (P<0.05). Treatment led to decreased TNF-α in both groups (P<0.05), though the viral group demonstrated a greater reduction (P<0.05, Table 4).

**Table 4:** Comparison of proinflammatory factors. Note: * indicates P < 0.05 compared with before-treatment.

		Bacterial group<br>(n=50)	Viral group (n = 50)	t	P
TNF-a (pg/mL)	Before treatment	85.23±5.94	71.02±9.88	8.716	<0.001
	After treatment	51.50±8.30*	31.58±7.31*	12.734	<0.001
	Before - after	33.73±8.89	39.44±12.52	2.630	0.010

### IgE variation analysis

Baseline IgE levels did not differ significantly between the viral and bacterial groups (P>0.05). After treatment, IgE decreased in both groups (P<0.05), but the difference between them remained non-significant (P>0.05). The pre- and post-treatment IgE variations were also comparable between groups (P>0.05, Table 5).

**Table 5:** Comparison of IgE.

		Bacterial group (n=50)	Viral group (n = 50)	t	P
IgE (IU/mL)	Before treatment	63.90±8.78	64.55±6.59	0.420	0.676
	After treatment	41.66±9.14	42.15±8.77	0.273	0.786
	Before - after	22.24±14.59	22.40±10.39	0.064	0.949

### Comparative analysis of IgG subclasses

At both baseline and after treatment, the viral group exhibited elevated IgG1 and IgG3 concentrations relative to the bacterial group (P<0.05), IgG2 levels were significantly reduced (P<0.05), while IgG4 showed no variation between groups (P>0.05). Compared to baseline, both groups had higher IgG1 level after treatment; however, the viral group displayed a larger IgG1 increase (P<0.05). Additionally, only the viral group showed elevated IgG3 post-treatment (P<0.05), with no significant change in the bacterial group (P>0.05). Neither group demonstrated significant alterations in IgG2 and IgG4 levels post-treatment (P>0.05, Table 6).

**Table 6:** Comparison of IgG subclasses. Note: * indicates P < 0.05 compared with before-treatment.

		Bacterial group (n = 50)	Viral group (n = 50)	t	P
IgG1 (mg/dL)	Before treatment	431.53±78.97	501.37±82.22	4.332	<0.001
	After treatment	522.47±89.18*	667.30±107.21*	7.344	<0.001
	Before - after	-90.95±118.07	-165.93±133.96	2.969	0.004
IgG2 (mg/dL)	Before treatment	356.19±67.81	302.38±61.20	4.166	<0.001
	After treatment	351.53±67.96	305.73±57.38*	3.641	<0.001
	Before - after	4.67±97.02	-3.35±82.99	0.444	0.658
IgG3 (mg/dL)	Before treatment	94.95±20.34	133.47±14.93	10.804	<0.001
	After treatment	96.43±18.89	152.31±16.78*	15.643	<0.001
	Before - after	-1.48±25.47	-18.84±22.30	3.626	<0.001
IgG4 (mg/dL)	Before treatment	82.39±12.86	82.57±10.04	0.079	0.938
	After treatment	83.29±11.51	82.16±9.27	0.540	0.590
	Before - after	-0.90±17.61	0.41±12.66	0.427	0.670

## Discussion

The immunopathological mechanisms underlying SA vary significantly depending on the causative pathogen, directly influencing clinical management strategies [12]. Consequently, characterizing immune profiles in SA cases triggered by distinct infections is critical for precise diagnosis and personalized therapy. This study identified key differences in serum inflammatory mediators and Ig levels between viral- and bacterial-induced SA in pediatric patients.

(i) Th1/Th17 co-activation inflammatory markers: The viral group showed elevated IFN-γ and IL-17A levels compared to the bacterial group, indicating a stronger Th1/Th17 co-activation immune response in viral infections. This aligns with the known mechanism where viruses trigger epithelial cells via PRRs, prompting IFN-γ release to eliminate viral particles [13]. Studies reveal that Th1/Th17 coactivation pathway hyperactivity is more effectively curbed by antiviral therapies like oseltamivir, as evidenced by significant drops in IFN-γ and IL-17A in viral cases [14]. For bacterial infections, IL-17A levels start lower and reduce more gradually post-treatment, possibly because of persistent neutrophil infiltration and IL-17A's involvement in tissue healing processes [15]. (ii) Th2 inflammatory markers: Compared to bacterial infections, viral infections showed lower baseline IL-4, IL-5, and IL-13 levels. This indicates that bacterial pathogens - possibly via LPS or peptidoglycan - trigger innate immunity, promoting Th2 cytokine secretion and worsening airway hyperreactivity [16]. Although corticosteroid treatment reduced Th2 cytokines in both groups, the viral group demonstrated a sharper decline, suggesting greater glucocorticoid sensitivity in virus-driven Th2 inflammation. (iii) Inflammatory mediators: Baseline and post-treatment TNF-α concentrations were elevated in bacterial infections relative to viral cases, potentially due to direct macrophage activation by bacterial endotoxins [17]. In contrast, the swift reduction in viral-associated TNF-α implies confined airway inflammation, while bacterial infections trigger prolonged systemic inflammation. (iv) Total IgE: SA is fundamentally an IgE-mediated Th2-type allergic disorder. Consequently, elevated baseline IgE levels were observed in both groups [18]. Both treatment groups showed only modest reductions in total IgE levels.

This limited decrease can be attributed to IgE's long half-life (approximately 2-3 weeks), making substantial short-term reductions unlikely [19]. Additionally, ongoing allergen exposure in SA patients may sustain elevated IgE levels due to incomplete allergic background control. (v) Ig subclasses: Elevated IgG1 (against viral protein antigens) and IgG3 (high-affinity complement activators) were observed in the viral group at baseline and post-treatment compared to the bacterial group, suggesting faster humoral immunity activation against viral infection. Higher IgG2 levels (against bacterial polysaccharides) were sustained in the bacterial group pre- and post-treatment compared to the viral group, implying prolonged B-cell activation by capsular antigens to improve phagocytosis [20]. At the same time, IgG1/IgG3 elevation in viral infections reflects Th1-polarized class switching promoting viral neutralization, while bacterial-driven IgG2 rise stems from Th2-induced B-cell activation against polysaccharide antigens [21]. Unchanged IgG4 levels could be attributed to the short treatment duration being insufficient to modify the underlying SA-related allergic state.

Based on our results, we suggest: (i) Standard bacterial cultures are time-intensive (24-48h), risking delayed care in SA cases. Implementing multiplex PCR for swift viral identification could prevent unnecessary antibiotic use. (ii) In pediatric viral infections, combining anti-IL-17 monoclonal antibodies or JAK inhibitors can help suppress excessive Th17 pathway activation. In cases of bacterial infections, extended antibiotic therapy is necessary, with IgG2 level monitoring to assess bacterial clearance efficacy, (iii) Tracking changes in the IgG1-to-IgG3 ratio aids in distinguishing between viral and bacterial infections, while sustained high IgG2 could suggest lingering bacterial antigens or inadequate therapy.

The current research has certain boundaries that warrant attention. (i) With merely 100 participants recruited from a single institution, the findings might not be broadly applicable; (ii) The analysis covered only solitary viral/bacterial infections; other infectious agents (fungi, mycoplasma) and polymicrobial infections were not examined immunologically. (iii) This analysis was restricted to acute/remission phase comparisons, lacking long-term follow-up (e.g., over 6 months) to assess how immunological markers relate to relapse risk. (iv) Concomitant therapies (e.g., corticosteroids) may produce biomarker reductions. However, due to the small number of cases in this study, subgroup analyses according to concomitant therapy are not available. Further analysis of this limitation is needed in future studies. Future multicenter studies with larger cohorts and extended observation periods are needed to address these limitations.

## Conclusion

In SA, viral infections drive Th1/Th17-dominant inflammation with IgG1/IgG3 elevation, enabling rapid neutralization but risking tissue damage. Bacterial infections promote persistent Th2 polarization and IgG2 dominance, delaying resolution. Testing IgG subclasses can reveal new insights for pathogen identification. Future research should integrate multi-omics technologies to clarify the interactions between pathogens, immunity, and inflammation, enabling precision medicine applications.

## Dodatak

### Availability of data and materials

The data used to support the findings of this study are available from the corresponding author upon request.

### Funding

The authors did not receive support from any organization for the submitted work.

### Acknowledgements

Not applicable.

### Conflict of interest statement

All the authors declare that they have no conflict of interest in this work.
